# Experiences of childhood emotional maltreatment and emotional intelligence in young women

**DOI:** 10.3389/fpsyt.2025.1583066

**Published:** 2025-07-04

**Authors:** Thomas Suslow, Anette Kersting, Dennis Hoepfel

**Affiliations:** Department of Psychosomatic Medicine and Psychotherapy, University of Leipzig Medical Center, Leipzig, Germany

**Keywords:** emotional neglect, emotional abuse, childhood maltreatment, trait emotional intelligence, emotion perception, emotion use, emotion understanding, emotion regulation

## Abstract

**Background:**

Research on the long-term consequences of childhood maltreatment underscores its contribution to impairments in cognitive-affective functions. According to trait models, emotional intelligence is subdivided into experiential abilities (emotion perception and thought facilitation through emotion) and strategic abilities (understanding and managing emotion). In the present study, we examined the relationship of emotional and other forms of childhood maltreatment with overall trait emotional intelligence and its specific facets in women with adverse childhood experiences.

**Materials and Methods:**

Our sample consisted of ninety-seven young women with experiences of childhood maltreatment as assessed with the Childhood Trauma Questionnaire (CTQ). Trait emotional intelligence was measured using the Self-Rated Emotional Intelligence Scale (SREIS). Participants’ verbal intelligence, cognitive flexibility, trait anxiety, and depressive symptoms were also assessed.

**Results:**

Emotional neglect was negatively correlated with the SREIS subscale Understanding emotions. Regression analysis indicated that emotional neglect was a significant predictor of understanding emotion independent of women’s verbal intelligence, cognitive flexibility, trait anxiety, and depressive symptoms. Neither emotional abuse nor other CTQ subscales showed correlations with any of the SREIS scores.

**Discussion:**

Experiences of emotional neglect during childhood but not of other types of maltreatment seem to go along with a decreased ability to understand and verbalize emotional states in adulthood. Thus, early emotional neglect could have an impact on strategic emotional abilities. Emotional neglect may have a greater effect on the development and expression of emotional intelligence than emotional abuse.

## Introduction

Child maltreatment can comprise acts of commission like emotional, physical, and sexual abuse and acts of omission like emotional and physical neglect by a parent or caregiver that result in harm or potential harm to a child’s development and health (1). Experiences of childhood maltreatment increase the risk for somatic and mental disorders (2, 3) and social dysfunction (4). Similar to traumatic experiences in adulthood (5) child abuse and neglect have massive, long-lasting consequences on adult psychopathology and treatment response (6) and augment risk for suicidality (7). Self-stigma, i.e., internalization of negative stereotypes about one’s experiences, mediates the relationship between childhood maltreatment and symptom levels of depression and anxiety (8). The experience of stigma has profound effects on mental health and well-being, in particular in women, who appear more vulnerable to discrimination compared to men (9). Child maltreatment is a widespread problem with far reaching ramifications for the child, and society at large. A recent study on the prevalence of childhood maltreatment in a nationwide young adult sample of the German population revealed that 18% of participants have experienced at least one type of maltreatment during their childhood (10). In the latter study, emotional neglect was reported most frequently (9%), followed by physical neglect (8.6%), emotional abuse (6.7%), physical abuse (3.7%), and sexual abuse (3.5%). Women are at a higher risk for sexual and emotional abuse during childhood compared to men (10, 11). Research on the short- and long-term consequences of child maltreatment underlines its contribution to impairments in cognitive-affective functions (12, 13). According to developmental models, emotional awareness undergoes development to a large extent within close attachment relationships (14). Early and enduring experiences of neglect and abuse can disturb the development of cognitive-affective competencies, resulting in difficulties in identifying and verbalizing emotions and an externally oriented thinking style, i.e., alexithymic personality characteristics (15, 16). Emotionally maltreated children lack positive models for developing emotional self-awareness and effective emotion regulation strategies (17). Results from recent meta-analyses indicate that emotional and physical neglect but also emotional abuse experienced in childhood are linked to high levels of alexithymia in adulthood (18, 19). Moreover, child maltreatment was found to be associated with poor emotion regulation as well as increased avoidance and emotional suppression in affected adults (20). There is evidence that emotional forms of childhood adversity (i.e., emotional abuse and emotional neglect) have more detrimental effects on socio-emotional functioning than physical or sexual abuse (21). Much of the previous research on cognitive-affective consequences of childhood maltreatment focused on alexithymic personality traits, and emotional dysregulation whereas the effects on emotional intelligence have received comparatively less attention.

The construct of emotional intelligence was developed and popularized in the 1990s (22, 23). Three main groups of emotional intelligence models can be distinguished based on the way the construct is assessed: performance-based ability, self-report trait, and self-report mixed models (24). In ability models, emotional intelligence is considered a type of aptitude or skill, and performance tasks are administered to measure it (25, 26). Ability-based test instruments of emotional intelligence are measures of maximal performance and indicate the objective ability to perceive and understand emotions. In contrast, trait models view emotional intelligence as typical behavior tendencies and self-perceptions regarding one’s ability to identify, use and control emotions (27, 28). Self-report instruments are administered to measure trait emotional intelligence. Finally, mixed models also measure emotional intelligence based on self-report, but their measurement construct is broader and comprises (among others) motivations, interpersonal abilities, personality factors, and empathy (29). Mayer and Salovey (30) divided emotional intelligence into an experiential area consisting of the ability to perceive emotions (emotion perception) and the capacity to use emotions to assist thinking and problem-solving (thought facilitation) and into a strategic area, which include the ability to understand emotional states, their causes, verbalization, and consequences (understanding emotion), and the ability to regulate emotional reactions in oneself and other people (managing emotion) (31). The abilities to perceive and use emotions are thought to reflect lower-order or rapid abilities in emotional processing (32, 33), whereas the strategic abilities to understand and regulate emotions are assumed to represent higher-order or deliberate processes of emotion control and emotional reasoning (33). Emotional intelligence is linked to better physical and mental health (34–37), heightened subjective wellbeing (38), and optimism (39). Emotional intelligence is discussed as a protective factor against anxiety (40) and depression (41). Individuals with high emotional intelligence could be better at controlling negative emotions and reducing stress reactions (42, 43). Identification and labeling of one’s own emotions helps people to automatically attenuate negative emotional experiences (44). The ability to recognize and share emotions of others strengthens social relationships and promotes quality of social interactions (45, 46). Abilities in regulating one’s emotions allow individuals to navigate difficult situations enhancing their resilience and self-esteem (47, 48).

Prior research on child maltreatment and emotional intelligence has centered around questions concerning the mediating role of emotional intelligence in the relationship of childhood maltreatment with depression and anxiety (49), psychological distress (50), life satisfaction (51), or cognitive emotion regulation strategies (52). There exists a research gap regarding the link between specific types of childhood maltreatment and specific facets of emotional intelligence. A secondary finding of previous research was (49–51) that overall childhood maltreatment severity showed negative correlations of small to medium size (*r* = -.20) with trait emotional intelligence (as assessed by the total score of the Wong Law Emotional Intelligence Scale (WLEIS (53)). Another investigation with a sample of teenagers (54) reported negative correlations of emotional abuse and emotional neglect with total trait emotional intelligence (*r*s = -.22 and -.37, respectively) (see also (55) for similar findings and (56) for null results related to ability and trait emotional intelligence). But this research based on samples of university or school students did not address the question of which types of childhood experiences are related to which facets of emotional intelligence.

In the present study, we examined the relationship of childhood maltreatment with trait emotional intelligence in a sample of women with various adverse childhood experiences. Women suffer childhood maltreatment more often than men. Based on previous results observed in samples of university students (54), we hypothesized that emotional abuse and emotional neglect would show negative correlations with trait emotional intelligence. The main aim of our study was to investigate which specific facets of emotional intelligence are associated with the emotional forms of childhood maltreatment. When analyzing the relationship between childhood maltreatment and emotional intelligence we controlled for participants’ verbal intelligence, cognitive flexibility, depressive symptoms and trait anxiety. Childhood maltreatment is known to be linked to negative affect (57, 58) and impairments in executive functions, processing speed, and verbal intelligence in adulthood (59, 60).

## Materials and methods

### Participants

Study participants were women recruited via public notices and online advertisements. We were looking for women who had suffered at least one form of abuse or neglect in their childhood. The main forms of possible abuse (emotional, physical, and sexual) and neglect (emotional and physical) were mentioned in our advertisements. The final sample consisted of 97 young women with a mean age of 23.2 years (SD = 3.4; range: 18–30). Most participants were university students (91%) from diverse academic disciplines. The other participants were in vocational training (n = 3) or working (n = 6). Interested individuals were interviewed via telephone to check criteria for in- and exclusion. To be included in the present study, participants had to have experienced at least one type of maltreatment during their childhood. Further inclusion criteria were native German language and age between 18 and 30 years. We restricted our sample to young adults to reduce the probability of memory inaccuracies concerning their maltreatment experiences during childhood. Exclusion criteria were the presence of a diagnosed mental disorder, use of psychotropic medication, and neurological diseases. Women with psychotherapeutic, psychiatric, or neurological treatments were excluded. The study procedure was approved by the relevant ethics committee of the University of Leipzig, Medical School (DE/EKSN40). Before the experiment, written informed consent was obtained. Participants were financially compensated for their involvement in the study.

### Questionnaires and tests

The Childhood Trauma Questionnaire (CTQ (61), German version (62)) was applied to assess traumatic childhood experiences. The CTQ comprises five subscales of childhood maltreatment: emotional abuse, physical abuse, sexual abuse, emotional neglect, and physical neglect. Each CTQ scale consists of 5 items, which are rated on a 5-point scale (from 1 = “applies not at all” to 5 = “applies entirely”). Subscale scores can range from 5 to 25, whereas total scores can range from 25 to 125, with higher values indicating more severe neglect or abuse. Bernstein and Fink (61) proposed four categories of severity for each trauma type and the total trauma experience: None (minimal); Low (to moderate); Moderate (to severe); and Severe (to extreme). In our study, Cronbach’s alpha was 0.81 for the total CTQ, 0.71 for emotional abuse, 0.80 for physical abuse, 0.85 for sexual abuse,.80 for emotional neglect, and 0.51 for physical neglect. Thus, internal consistencies were satisfactory for the CTQ scales with the exception of physical neglect. A prior study on the validity of the German adaptation of the CTQ (63) reported also a low internal consistency of the subscale physical neglect.

To measure emotional intelligence, we administered the *Self-Rated Emotional Intelligence Scale* (SREIS (64), German version (65)). SREIS assesses four facets or domains of emotional intelligence: perception, use, understanding, and regulation of emotion. The SREIS comprises five subscales as the facet *emotion regulation* is subdivided into the regulation of one’s own emotions (Managing emotion (self)) and regulating emotions in other people (Social management). The scale Perceiving emotion consists of four items related to the perception and correct identification of emotions expressed by other people (e.g., “By looking at people’s facial expressions, I recognize the emotions they are experiencing”). The scale Use of emotion has three items related to the ability to harness emotions, which can assist reasoning and problem-solving processes (e.g., “When making decisions, I listen to my feelings to see if the decision feels right”). The scale Understanding emotion comprises four items that relate to the emotional vocabulary, the ability to verbalize and understand emotions (e.g., “I have the vocabulary to describe how most emotions progress from simple to complex feelings”). The scale Managing emotion (self) has four items relating to the ability to change or control one’s own emotional responses (e.g., “I am able to handle most upsetting problems”). Finally, the scale Social management consists of four items, which refer to the ability to regulate emotions in other persons (e.g., “When someone I know is in a bad mood, I can help the person calm down and feel better quickly”). By summing the subscale scores of the SREIS a total emotional intelligence score can be computed. The items of the SREIS are rated on a Likert-type scale (from 1 (inaccurate) to 5 (accurate)). In the present study, Cronbach’s alpha was 0.81 for Perceiving emotion, 0.83 for Use of emotion, 0.84 for Understanding emotion, 0.67 for Managing emotion (self), 0.68 for Social management, and 0.80 for the total SREIS score.

The *Beck Depression Inventory* is a multiple-choice self-report scale (BDI-II (66); German version (67)) that measures cognitive, affective, and neurovegetative symptoms of depression such as negative cognitions, hopelessness, sadness, and physical symptoms during the preceding two weeks. The BDI-II consists of 21 items. Questions have a set of four possible answer choices, ranging in intensity. Total scores of the BDI-II range from 0 to 63. In our sample, Cronbach’s alpha for the BDI-II was 0.87.

The *State-Trait Anxiety Inventory* (STAI (68); German version (69)) is a self-report questionnaire assessing state and trait anxiety. The STAI comprises 20 items, respectively, which are rated on a 4-point scale. The trait version of the STAI assesses stable interindividual differences in experiencing and evaluating situations as threatening. In our study, only the trait version of the STAI was administered. Cronbach’s alpha for the STAI trait was 0.90 in the present sample.

The *Multiple-choice vocabulary intelligence test* (Mehrfachwahl-Wortschatz-Intelligenztest, MWT-B (70)) is a performance test without time restrictions, which measures verbal intelligence. The MWT-B consists of 37 items. Each item comprises one real German word and four pronounceable pseudo-words. Participants are asked to find the correct word and to underline it. Each word correctly identified gives a point, which is added to the total score for a maximum of 37. Based on data from a representative sample MWT-B raw scores can be converted to IQ scores (70).

Part B of the *Trail Making Test* (TMT-B (71)) was administered to assess cognitive flexibility. In this test, participants´ task is to connect numbers and letters in ascending order. The total time needed for task completion serves as an indicator of cognitive flexibility.

### General procedure

The individual testing sessions took place in a quiet room at the Department of Psychosomatic Medicine and Psychotherapy at the University of Leipzig. Study participants were examined between May and December 2023. At the beginning of the study, participants were informed that the investigation aimed at examining maltreatment experiences during childhood and current perception of and attention to emotions. Study participants were blind to research hypotheses. After completing a sociodemographic questionnaire, a series of tests and questionnaires were administered in a fixed order: CTQ, BDI-II, STAI, MWT-B, SREIS, and TMT-B. The administration of these test instruments took about 45 minutes.

### Statistical analysis

Shapiro-Wilk tests were applied to assess the normality of distribution. Since violations of the assumption of normal distribution were found in the majority of the variables examined in our study (*p*s < 0.05; except for the CTQ total scale, SREIS total scale, and SREIS subscales understanding emotion and social management) we administered mainly nonparametric statistical tests. The Friedman test was used to analyze differences in severity of childhood maltreatment types reported in our sample. Wilcoxon tests were used as *post hoc* tests. Spearman rank correlation was used to examine the relationships between childhood maltreatment types, facets of emotional intelligence, verbal intelligence, cognitive flexibility, depression, and trait anxiety in our sample. In the first step, it was tested whether emotional abuse and emotional neglect are correlated with (total) emotional intelligence. In the second step, we examined whether emotional abuse and emotional neglect are correlated with specific facets of emotional intelligence. This represents the main research question of our study. To correct multiple testing, we used an adjusted p-level of 0.005 (two-tailed) in the correlation analyses between CTQ and SREIS, i.e., we divided the standard significance level of *p* = 0.05 by ten (2 emotional forms of childhood maltreatment x 5 facets of emotional intelligence). The majority of correlation analyses were conducted to control confounders and to identify factors associated with childhood maltreatment or facets of emotional intelligence. In addition, hierarchical regression analysis was performed for those facets of emotional intelligence, which showed correlations with childhood maltreatment, to examine whether these relationships remain significant after adjusting the effects of other relevant variables, i.e., verbal intelligence, cognitive flexibility, depression, and trait anxiety. Regression analysis is robust against non-normality but regression residuals should follow a normal distribution. We controlled multicollinearity of variables using variance inflation factor and tolerance (72). The Durbin-Watson test was applied to test for serial autocorrelation. Durbin-Watson values around 2 mean that there is no serial autocorrelation. Statistical analyses were made with SPSS software version 29.0 (IBM Corp., Armonk, NY, USA).

*A priori* analysis of statistical power was computed with the program G*Power (version 3.1.9.2.; bivariate normal model - exact test family) of Faul et al. (73). To detect a medium effect of *r* = 0.30 with an alpha value of 0.05, two-tailed, and a power of 0.80 the required total sample size is 84. Note that Ren et al. (54) reported a correlation of *r* = -.37 between emotional neglect (assessed with a short form of the CTQ (74) and emotional intelligence (total score of the WLEIS (53)) in a Chinese sample.

## Results

### Severity of childhood maltreatment

According to the Friedman test, there were significant differences in severity between childhood maltreatment types in our sample, χ^2^ (4) = 280.95, *p* < 0.001 (descriptive statistics for CTQ data are shown in **Table 1**). The results of Wilcoxon tests indicated that severity of all childhood maltreatment types differed significantly from each other (emotional neglect > emotional abuse > physical neglect > physical abuse > sexual abuse, *p*s <.01). In addition, we classified study participants into the four categories of severity for each trauma type and the total trauma experience as proposed by Bernstein and Fink (61) (see **Supplementary Table 1**). When considering the total CTQ score, all participants were characterized by childhood maltreatment experience - at least of low severity. More than half of our participants reported to have experienced severe emotional neglect (66%) or severe emotional abuse (54%). In contrast, more than half of our sample reported no experiences of physical abuse (65%) or sexual abuse (68%). For physical neglect, the distribution of scores was more evenly distributed across severity categories (see for details **Supplementary Table 1**).

**Table 1:** Descriptive statistics of and Spearman rank correlations between CTQ scales and SREIS scales (N = 97).

Variable	CTQ total	CTQ EA	CTQ PA	CTQ SA	CTQ EN	CTQ PN	*Mean*	*SD*
SREIS total	-.08	-.08	.08	.10	-.20	-.04	64.03	9.62
PE	.01	-.03	.04	.10	-.05	.06	15.51	3.01
UsE	-.09	-.06	.10	.10	-.21	-.12	10.58	3.17
UnE	-.19	-.14	.06	.01	-.29*	-.14	12.52	3.76
ME	.01	-.05	-.01	.00	.04	.05	10.82	3.29
SM	.05	.09	.16	.07	-,10	-.01	14.61	2.86
*Mean*	58.89	15.86	7.88	6.71	18.47	9.97		
*SD*	11.10	4.25	4.00	3.60	3.57	2.96		

*p <.005 (two-tailed).

CTQ, Childhood Trauma Questionnaire; CTQ-EA, scale emotional abuse; CTQ-PA, scale physical abuse; CTQ-SA, scale sexual abuse; CTQ-EN, scale emotional neglect; CTQ-PN, scale physical neglect; SREIS, Self-Rated Emotional Intelligence Scale; PE, perceiving emotion scale; UsE, use of emotion scale; UnE, understanding emotion scale; ME, managing emotion (self) scale; SM, social management scale.

### Relationships between CTQ, SREIS, MWT-B, TMT-B, BDI-II, and STAI

Means with standard deviations for SREIS are presented in **Table 1**. Emotional neglect (CTQ) was negatively correlated with the SREIS subscale Understanding emotion (see also **Supplementary Figure 1** for a plot illustrating the correlation between emotional neglect and understanding emotion). This correlation was of medium size. No other significant correlations were observed between the scales of the CTQ and the SREIS.

Means with standard deviations for MWT-B, TMT-B, BDI-II, and STAI are presented in **Table 2**. Emotional abuse (CTQ) was positively correlated with the STAI. There were no other significant correlations of the CTQ with the MWT-B, TMT-B, BDI-II, or STAI.

**Table 2:** Descriptive statistics of and Spearman rank correlations between CTQ scales and other self-report scales (BDI-II and STAI) and tests (MWT-B and TMT-B) (N = 97).

Variable	CTQ total	CTQ EA	CTQ PA	CTQ SA	CTQ EN	CTQ PN	*Mean*	*SD*
MWT-B IQ	-.11	-.13	-.15	.06	-.07	-.05	106.30	9.47
TMT-B	.13	.02	.12	.10	.07	.10	65.51	26.15
BDI-II	.19	.16	.07	-.02	.17	.16	16.42	9.02
STAI	.19	.25*	.02	.01	.14	.16	50.12	10.09
*Mean*	58.89	15.86	7.88	6.71	18.47	9.97		
*SD*	11.10	4.25	4.00	3.60	3.57	2.96		

*p <.05 (two-tailed).

CTQ, Childhood Trauma Questionnaire; CTQ-EA, scale emotional abuse; CTQ-PA, scale physical abuse; CTQ-SA, scale sexual abuse; CTQ-EN, scale emotional neglect; CTQ-PN, scale physical neglect; MWT-B IQ, Multiple-choice vocabulary test version B, intelligence quotient; TMT-B, Trail-Making-Test version B; BDI-II, Beck Depression Inventory; STAI, State-Trait Anxiety Inventory, trait version.

The SREIS total score was positively correlated with the MWT-B and negatively correlated with the BDI-II and STAI (see **Table 3**). The SREIS subscales Perceiving emotion, Use of emotion, and Social management showed no correlations with the MWT-B, TMT-B, BDI-II, and STAI. The subscale Understanding emotion was positively correlated to the MWT-B and negatively correlated with the BDI-II and STAI. The subscale Managing emotion (self) was negatively correlated with the BDI-II and STAI.

**Table 3:** Spearman rank correlations between SREIS and other self-report scales (BDI-II and STAI) and tests (MWT-B and TMT-B) (N = 97).

Variable	SREIS total	SREIS PE	SREIS UsE	SREIS UnE	SREIS ME	SREIS SM
MWT-B IQ	.21*	.05	.09	.22*	.05	.11
TMT-B	.09	.08	.11	-.01	.01	.12
BDI-II	-.31**	.11	-.07	-.33**	-.43***	-.13
STAI	-.30**	.14	-.03	-.30**	-.56***	-.07

*p <.05 (two-tailed), **p <.01 (two-tailed), ***p <.001 (two-tailed).

SREIS, Self-Rated Emotional Intelligence Scale; PE, perceiving emotion scale; UsE, use of emotion scale; UnE, understanding emotion scale; ME, managing emotion (self) scale; SM, social management scale; MWT-B IQ, Multiple-choice vocabulary test version B, intelligence quotient; TMT-B, Trail-Making-Test version B; BDI-II, Beck Depression Inventory; STAI, State-Trait Anxiety Inventory, trait version.

A regression model for understanding emotion was calculated to examine whether emotional neglect is a predictor independent from verbal intelligence (MWT-B), cognitive flexibility (TMT-B), depressive symptoms (BDI-II), and trait anxiety (STAI). The initial model did not significantly predict understanding emotion (see **Table 4**). In step two entering emotional neglect significantly increased the predictive value of the model. This means, emotional neglect was found to be a significant negative predictor of understanding emotion. When considering the standardized beta value (-0.222) the size of the effect was rather small. The regression residuals of the model were normally distributed (Shapiro-Wilk *W* = 0.98, df = 97, *p* > 0.05). A Q-Q plot of the standardized residuals concerning the regression model is shown in **Supplementary Figure 2**. The Durbin-Watson test yielded *d* = 1.80. VIF and tolerance values indicate no multicollinearity issues in this regression model.

**Table 4:** Hierarchical regression predicting understanding emotion (SREIS) in two steps by verbal intelligence (MWT-B IQ), cognitive flexibility (TMT-B), depressive symptoms (BDI-II), trait anxiety (STAI), and emotional neglect (CTQ) (N = 97).

Step	Predictor	Coefficients			Multicollinearity Model				
		β [with 95% confidence interval]	Beta	*T*	Sig. (*p*)	Tol.	VIF	R^2^	ΔR^2^
Step 1	MWT-B IQ	.053 [-0.025,0.131]	.133	1.34	.183	.96	1.04	.135	–
	TMT-B	.001 [-0.027,0.029]	.006	0.06	.949	.99	1.01		
	BDI-II	-.113 [-0.241,0.014]	-.272	-1.76	.081	.39	2.53		
	STAI	-.028 [-0.143,0.087]	-.075	-0.48	.631	.39	2.58		
Step 2	MWT-B IQ	.054 [-0.022,0.131]	.136	1.40	.164	.96	1.04	.182	.047*
	TMT-B	.004 [-0.024,0.031]	.024	0.25	.799	.98	1.02		
	BDI-II	-.101 [-0.227,0.025]	-.242	-1.60	.113	.39	2.55		
	STAI	-.020 [-0.133,0.093]	-.054	-0.36	.723	.39	2.59		
	CTQ EN	-.235 [-0.439,-0.030]	-.222	-2.28	.025*	.94	1.06		

β, unstandardized regression coefficient; Tol., Tolerance; VIF, Variance Inflation Factor.

*p ≤.05 (two-tailed).

## Discussion

The purpose of this study was to investigate the relationships of childhood maltreatment types with facets of trait emotional intelligence in adulthood. To this aim, we recruited a sample of young adult women with various adverse childhood experiences. Prior research in the field recruited *ad-hoc* samples of school or university students (49–51, 54–56). Considering the means of the CTQ scales the women of our sample were primarily characterized by experiences of emotional neglect and emotional abuse during childhood, followed by physical neglect. In contrast, the CTQ scores for physical abuse and sexual abuse were comparatively low. According to the severity classification of Bernstein and Fink (61) many of our participants reported to have experienced severe emotional neglect or severe emotional abuse, whereas only quite a few reported experiences of severe physical abuse, severe sexual abuse, or severe physical neglect (see **Supplementary Table 1**). These maltreatment characteristics of our sample are largely consistent with the frequencies of maltreatment types in a recent nationwide young adult sample of the German population (5). In that study, young adults reported emotional neglect most frequently, followed by physical neglect, and emotional abuse.

Based on previous results (54) we hypothesized that emotional abuse and emotional neglect are negatively correlated with trait emotional intelligence. The present results suggest that neither experiences of emotional neglect nor experiences of emotional abuse during childhood are related to overall emotional intelligence. To investigate whether and which specific facets of emotional intelligence are associated with the emotional and other forms of childhood maltreatment we conducted additional correlation analyses at the subscale level of the SREIS. According to our results, emotional abuse was not linked to any of the emotional intelligence domains. In contrast, we observed that emotional neglect was significantly related to the understanding of emotion (see **Supplementary Figure 1**). As indicated by our regression analysis, this relation was independent of women’s trait anxiety, depressive symptoms, verbal intelligence, and cognitive flexibility. No correlations were found between emotional neglect and perceiving emotion, using emotion, and (intrapersonal and interpersonal) emotion regulation. In our study, there was no association between overall childhood maltreatment severity and overall trait emotional intelligence, which is not consistent with previous findings showing a moderate negative correlation between these variables (49–51). One explanation for the different correlation results could be differences in the severity of childhood maltreatment, which was substantially lower in other investigations (49–51) compared to our study.

In the present study, overall emotional intelligence as well as the facet understanding emotion were both positively related to verbal intelligence and negatively related to the level of depressive symptoms and trait anxiety. These findings indicate that the ability to understand one’s emotions might have mood-protective effects. As mentioned in the introduction, emotional intelligence is assumed to be a protective factor against anxiety and depression (40, 41), which could also promote the prevention and reduction of stress symptoms (42).

We found evidence that experiences of emotional neglect during childhood (but not of other types of maltreatment) go along with a reduced ability to understand emotional states and to verbalize them. These data suggest that early experiences of emotional neglect, i.e., having received no or little emotional attention and response from caregivers could be linked to a restricted vocabulary to describe one’s emotions in adulthood. Thus, emotional neglect in childhood might have effects on a strategic area (i.e., emotion understanding) of later emotional intelligence. This means that early experiences of emotional neglect could affect higher-order cognitive-affective processes. According to our data, emotional neglect may have a greater impact on the development and expression of emotional intelligence than emotional abuse. The unavailability of positive role models in the area of ​​emotion-related learning during childhood could have more negative effects on the development of conceptual emotion knowledge than models that control their children by using emotions to frighten, embarrass, blame, or otherwise manipulate them. In the latter case, an emotional exchange still occurs (albeit highly problematic) through which emotions of various quality may become objects of experience and regulation. Our results underline that childhood emotional neglect although it appears to be a “silent” form of maltreatment could have significant and lasting impacts on emotional competencies in adulthood. The present findings point to the importance that emotional care and attention received from caregivers during childhood may have for the development of higher-order processes of emotional reasoning. Experiences of early emotional neglect may affect the development of lower-order processes of emotion perception to a lesser extent than that of higher-order processes. The ability to perceive and recognize facial emotions in others relies, at least in part, on genetically determined processes of automatic mimicry (75) and internal simulation of the perceived emotional state of the other (76, 77). These basic neurobiological processes of acquiring information about others’ feelings appear less dependent on social learning than the development of linguistic knowledge, the vocabulary to verbalize one’s emotions. It is somewhat surprising, however, that we did not find any correlations between emotional neglect and emotion regulation abilities, which are also part of the strategic emotional intelligence facet. Possibly, emotional neglect by caregivers during childhood has a smaller negative effect on the development of emotion regulation skills than on the development of emotion understanding. There is evidence that peers - and especially friends - play a critical role in shaping and managing emotions during adolescence (78). Thus, peer relationships in adolescence may at least partially compensate for the negative effects of family or parental emotional neglect during childhood.

In the present study, we focused exclusively on trait emotional intelligence, i.e., typical behaviors and self-perceptions concerning one’s ability to recognize, use and regulate emotions (27, 28). Trait emotional intelligence is only weakly linked to ability emotional intelligence (79), which is assessed by measures of maximal performance and indicates the objective ability to perceive and understand emotional states. Against this background, it is an important task for future research to specifically examine the relationship between childhood maltreatment and emotional intelligence as assessed by ability tests.

The occurrence of neglect and the harm it does to children might be avoided or reduced through early prevention and intervention programs. As the effect of neglect appears especially damaging during infancy, it seems important to work with families as early as possible (80). Parent training programs can improve parents’ competencies and teach them to interact positively with their children, which includes contingent responsivity, sensitivity, and emotional communication (81). Neglected children might also be directly supported through interventions that improve social-emotional development of preschool children (82). For psychiatric patients with pronounced experiences of emotional neglect our findings may have potential therapeutic implications. It could be useful to offer these patients training in emotional skills, in particular concerning the abilities to recognize and verbally express emotions. One possible training program is the Cognitive Remediation and Emotional Skills Training (CREST) (83, 84), which has been found to improve identification of and attention to one’s feelings in patients suffering from eating disorders (85). Before implementing training measures, it seems advisable to control in each individual case whether a patient actually shows or reports impairments in identifying and verbalizing emotions.

Limitations of our study are that childhood maltreatment was retrospectively assessed by self-report and that the investigation was cross-sectional and not longitudinal in design. However, retrospective reports in adulthood of major adverse experiences in childhood are assumed to include only a low rate of false positive reports (86). Moreover, longitudinal data suggest that the CTQ provides temporally stable self-reports of childhood maltreatment in healthy and clinical populations (87). Nevertheless, there is a risk of recall bias in individuals with experiences of childhood maltreatment. Negative recall bias and faster access to negative autobiographical information has been found to be associated with tendencies to experience negative affect (88). As individuals with adverse childhood experiences are frequently characterized by increased negative affect, they might more frequently rehearse and more easily recall negative events and memories, which could lead to an over-reporting of childhood maltreatment (89, 90). The generalizability of our results is limited by the fact that we only included young women who experienced at least one type of child maltreatment in their lives. Exclusion of women with mental health disorders and use of psychotropic medication also limits the generalizability of our findings to women with a history of adverse childhood experience. Our findings concerning the CTQ subscale physical neglect must be interpreted with caution since the internal consistency of this subscale was low. The CTQ is only a brief screening tool for childhood maltreatment in adults, which does not assess exact intensity, duration, frequency, and age of first exposure to childhood maltreatment. Test instruments like the Maltreatment and Abuse Chronology of Exposure (MACE) scale provide more detailed information on timing of exposure to adverse childhood experience (91) and should therefore be used preferentially in future studies. We assessed cognitive flexibility using the TMT-B, which represents a limitation of our study because multimethod approaches have been recommended to capture its multiple components (92). It can also be criticized that the five types of childhood maltreatment examined varied in severity in our sample with emotional neglect and emotional abuse being the most prevalent. Future research is necessary to clarify the association of maltreatment experiences in childhood with facets of emotional intelligence in men. Future investigations may consider social support and resilience factors as moderating variables, which could affect the strength of the relationship between adverse childhood experience and emotional intelligence. Although in the present study we found no direct relations between emotional childhood maltreatment and several facets of emotional intelligence (except emotion understanding), it would be important to examine whether affective factors such as trait anxiety could act as mediating variables in some relationships.

## Data Availability

The raw data supporting the conclusions of this article will be made available by the authors, without undue reservation.
