# Supplementary material for: Experiences of childhood emotional maltreatment and emotional intelligence in young women
Source: Front Psychiatry. 2025 Jul 4;16:1583066. doi: 10.3389/fpsyt.2025.1583066 (PMC12271162; doi:10.3389/fpsyt.2025.1583066)
Supplement: Supplementary file 1 [file Table1.docx]

Supplementary Table 1: Distribution of study participants’ CTQ subscale and total scores ​​across categories of childhood maltreatment severity (classification as proposed by Bernstein and Fink, 1998).

| CTQ | None | Low | | Moderate | Severe |
| --- | --- | --- | --- | --- | --- |
| Emotional abuse | 3 | 21 | 21 | | 52 |
| Physical abuse | 63 | 11 | 10 | | 13 |
| Sexual abuse | 66 | 13 | 8 | | 10 |
| Emotional neglect | 2 | 11 | 20 | | 64 |
| Physical neglect | 18 | 31 | 32 | | 16 |
| Total CTQ | 0 | 27 | 52 | | 18 |
|  | | | | | |
