# Supplementary figures and images for: Experiences of childhood emotional maltreatment and emotional intelligence in young women
Source: Front Psychiatry. 2025 Jul 4;16:1583066. doi: 10.3389/fpsyt.2025.1583066 (PMC12271162; doi:10.3389/fpsyt.2025.1583066)

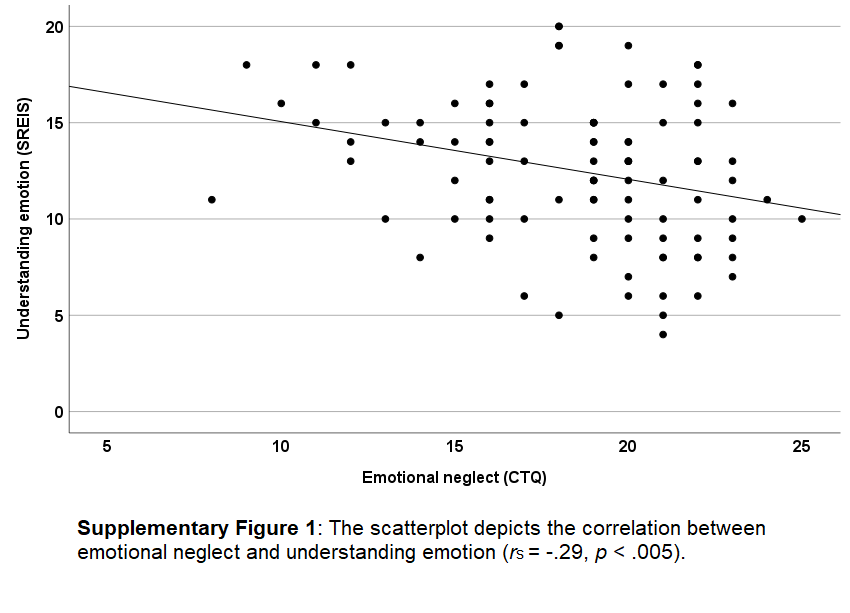

Supplement: Supplementary file 2 [file Image1.tif]

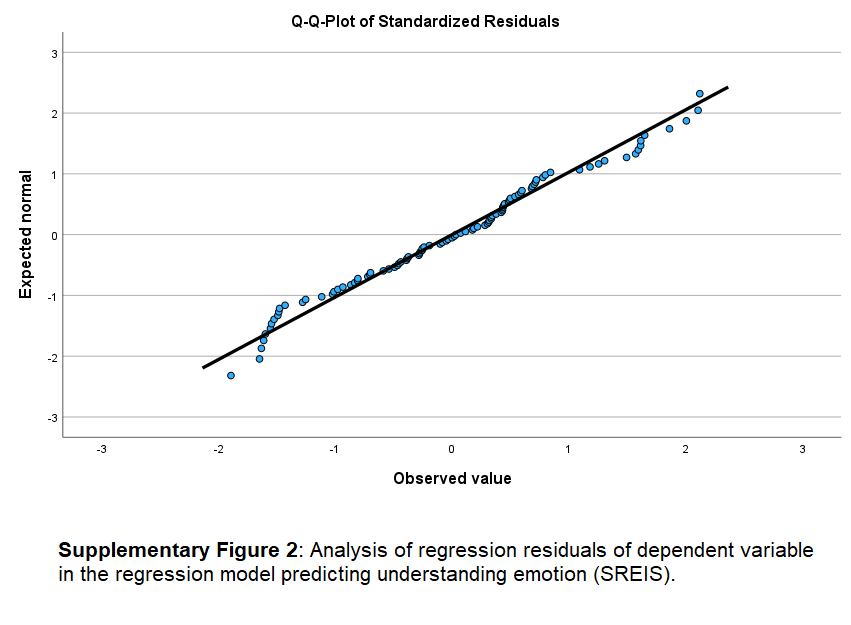

Supplement: Supplementary file 3 [file Image2.tif]
